# Data on natural radioactivity and associated radiation hazards in coastal sediment of Chennai Coast, Tamilnadu, India using gamma ray spectrometry

**DOI:** 10.1016/j.dib.2018.01.036

**Published:** 2018-02-02

**Authors:** M. Tholkappian, Durai Ganesh, E. Devanesan, N. Harikrishnan, J. Prince Prakash Jebakumar, R. Ravisankar

**Affiliations:** a Department of Physics, Sri Vari College of Education, Then Arasampattu, Tiruvannamalai 606611, Tamilnadu, India; b Post Graduate and Research Department of Physics, Government Arts College, Tiruvannamalai 606603, Tamilnadu, India; c Department of Physics, Divya Arts & Science College, Tiruvannamalai 606801, Tamilnadu, India; d Coastal and Environmental Engineering, National Institute of Ocean Technology, Pallikaranai, Chennai 600100, Tamilnadu, India

**Keywords:** Sediment, East coast of Tamil Nadu, Natural radionuclides, Gamma ray spectrometer, Radiation hazards

## Abstract

This article contains data on the activity concentration of natural radionuclides in coastal sediment samples collected from Pulicat Lake to Vadanemmeli, East coast of Tamil Nadu using NaI(Tl) detector based γ-spectrometry. As marine sediments are found to be the repository of many radioactive pollutants, studied the objectives like (i) determine natural radionuclide activity concentrations in sediment samples in and around Chennai coast (ii) evaluate the radiological hazards due to natural radioactivity associated with coastal sediments and (iii) identify areas which may be radiological hazardous for the public along the study area. The average activity concentration of ^238^U, ^232^Th and ^40^K in the present study is lower than world median value. The radiological hazard indices such as External hazard index (*H*_ex_) and Gamma representative level index, (Iγ) were evaluated to assess radiation hazard associated with the sediments. The simulated results show sediments do not pose any significant radiation hazards due to concentration of natural radionuclides.

**Specifications Table**

**Table:** 

Subject area	Physics
More specific subject area	Radioactivity and Radiation Hazards
Type of data	Table
How data was acquired	NaI (Tl) detector (Scionix make) based gamma spectrometer of 3′′ dia × 3′′ thick housed inside 15 cm thick Pb shielding with graded lining
Data format	Raw data
Experimental factors	The sediment samples were collected from Pulicat Lake to Vadanemmeli of Chennai Coast along the Bay of Bengal Coastline in Southeastern India using a Peterson grab sampler during the pre-monsoon condition. The grab sampler collects the samples at 10 cm below the seabed in all sampling points. The collected samples were immediately transferred to polythene bags in order to avoid the sediment samples contact with the metallic dredge and the top sediment layer was scooped with an acid washed plastic spatula. Samples were stored in plastic bags and kept in refrigeration at 4 °C until analysis. Then the samples were air dried at 105 °C for 24 h to a constant weight and sieved through 250 μm mesh. Sediment samples were subjected to gamma spectral analysis with a counting time of 20,000 s. The concentrations of various radionuclides of interest were determined in Bq kg^−1^ using the count spectra.
Experimental features	The activity concentrations of ^238^U, ^232^Th and ^40^K in sediment were determined using NaI(Tl) detector. The measurement for the natural radioactive elements ^40^K, uranium and thorium, the gamma energies selected are 1460 keV for ^40^K, 1763 keV (from daughter product ^214^Bi) for uranium and 2614 keV (from daughter product ^208^Tl) for thorium. The detection limit of NaI(Tl) detector system for ^40^K, ^238^U and ^**232**^Th are 8.50, 2.21 and 2.11 Bq kg^**–1**^ respectively for a counting time of 20,000 s.
Data source location	Pulicat Lake to Vadanemmeli, East Coast of Tamilnadu, India
Data accessibility	Data is with this article.

**Value of the data**

• This data information provides the natural radioactivity concentration in the coastal sediment samples of Chennai Coast, Tamilnadu, India.
• Data can be used as a base-line data for radionuclide concentration levels in marine environments.
• The data can be useful for other researchers investigating the assessment of radiation hazards
• Data provide baseline radiometric data on environmental radioactivity in the region for future epidemiological studies and environmental monitoring initiatives in the study area.

## 1 Data

### 1.1 Activity concentrations of ^238^U, ^232^Th and ^40^K in the sediments

**Fig. 1:**
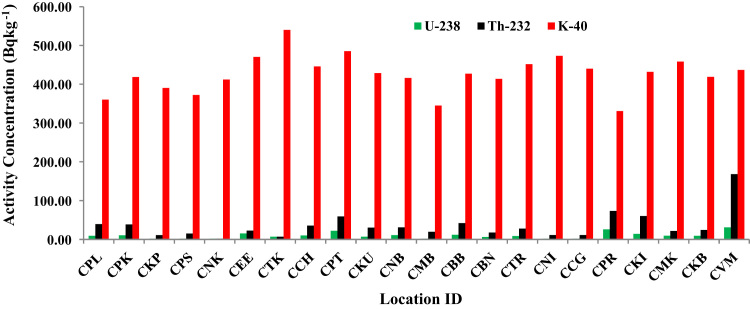
Location ID Vs Activity Concentration (Bq kg^−1^).

The activity concentrations of ^238^U, ^232^Th and ^40^K in sediment samples are given in Table 1. All values are given in Bq kg^−1^of dry weight. The range of activities and mean values (in brackets) for ^238^U, ^232^Th and ^40^K are ≤ 2.21 (BDL) – 31.03 (10.14), ≤ 2.11 (BDL) – 168.4 (35.02) and 330.91 – 540.02 (425.82) Bq kg^−1^ respectively. The wide variations of the activity concentration values are due to the presence of physical, chemical and geochemical properties of sediment in marine environment [1], [2]. The results show that the mean activity of ^238^U, ^232^Th and ^40^K are lower than the world average values (35 Bq kg^−1^ for ^238^U, 30 Bq kg^−1^ for ^232^Th and 400 Bq kg^−1^ for ^40^K) [3]. Fig. 1 shows the variation of activity concentration with sampling locations.

**Table 1:** Activity Concentration (Bq kg^-1^), External Hazard Index (H_ex_) and Gamma Representative level index (Iγ) of Coastal sediment samples of Chennai Coast, Tamilnadu, India.

**S. No**	**Name of the Location**	**Sample ID**	**Activity Concentration**			**External Hazard Index (H**_**ex**_**)**	**Gamma Representative level index** (**I**γ)
			^**238**^**U Bq kg**^**-1**^	^**232**^**Th Bq kg**^**-1**^	^**40**^**K Bq kg**^**-1**^		
1	Pulicat Lake	CPL	9.30	39.41	360.26	0.2528	0.6963
2	Pulicat	CPK	10.50	38.67	418.46	0.2653	0.7357
3	Kattupalli	CKP	2.21	10.98	390.51	0.1297	0.3849
4	Powerstation	CPS	2.21	14.96	372.54	0.1414	0.4127
5	Nettukuppam	CNK	2.21	2.11	412.09	0.0998	0.3106
6	Ennore	CEE	15.35	22.68	470.34	0.2272	0.6427
7	Tiruchinnakuppam	CTK	7.07	7.16	540.02	0.1591	0.4787
8	Chennai Harbour	CCH	10.04	35.70	445.85	0.2582	0.7212
9	Chennai Port	CPT	22.24	59.14	485.12	0.3902	1.0631
10	Kasimedu-Tondiarpet	CKU	7.09	30.39	428.69	0.2261	0.6370
11	Neppiar Bridge	CNB	11.10	30.94	416.12	0.2364	0.6608
12	Marina Beach	CMB	2.21	19.66	345.10	0.1539	0.4414
13	Broken Beach	CBB	12.04	41.98	427.33	0.2841	0.7850
14	Besent Nagar	CBN	6.48	17.72	413.87	0.1722	0.4963
15	Thiruvanmiyur	CTR	8.64	27.84	451.55	0.2251	0.6370
16	Neelankarai	CNI	2.21	11.54	473.13	0.1491	0.4456
17	Chennai Golden Beach	CCG	2.21	11.54	439.77	0.1421	0.4233
18	Panaiyur	CPR	25.98	73.41	330.91	0.4235	1.1279
19	Kanathursunami Area	CKI	14.13	60.15	431.85	0.3611	0.9836
20	Muttukaadu	CMK	9.56	21.74	458.54	0.2054	0.5868
21	Kovalam Beach	CKB	9.29	24.23	419.10	0.2062	0.5836
22	Vadanemmeli	CVM	31.03	168.40	436.99	0.8274	2.1822
	**Average**		**10.14**	**35.02**	**425.82**	**0.2517**	**0.7016**

### 1.2 Evaluation of radiological hazard effects

#### 1.2.1 External hazard index (*H*_ex_)

**Fig. 2:**
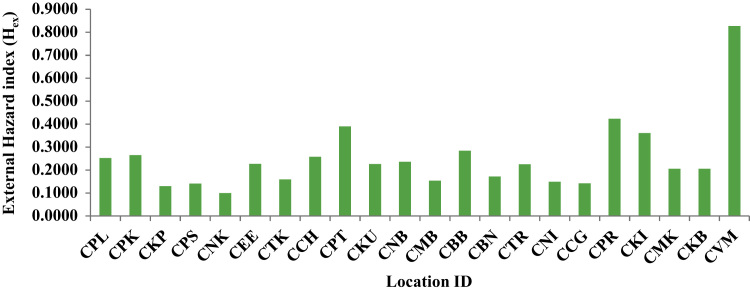
Location ID Vs External Hazard Index (*H*_ex_).

The external hazard index (*H*_ex_) represents the external radiation exposure associated with gamma radiation from radionuclides of concern. This index can be evaluated using the following equation [4], [5].

(1) $$H_{\mathrm{ex}}=\frac{A_{U}}{370\mathrm{Bq}/\mathrm{Kg}}+\frac{A_{\mathrm{Th}}}{259\mathrm{Bq}/\mathrm{Kg}}+\frac{A_{K}}{4810\mathrm{Bq}/\mathrm{Kg}}\leq 1$$

where *A*_U_, *A*_Th_ and *A*_K_ are the specific activities of ^238^U, ^232^Th and ^40^K in Bq kg^−1^ respectively. The value of *H*_ex_ must be lower than unity in order to keep the radiation hazard insignificant. Using the above formula *H*_ex_ had been estimated and tabulated in Table 1. The *H*_ex_ values ranged from 0.0998(Nettukuppam) to 0.8274(Vadanemmeli) with an average value of 0.2517, so the samples meet the condition *H*_ex_< 1.This implies that activities involving the use of sediments samples are safe and do not attract any high levels of radiation exposure. Fig. 2 shows the locations and *H*_ex_.

#### 1.2.2 Gamma representative level index, (Iγ)

**Fig. 3:**
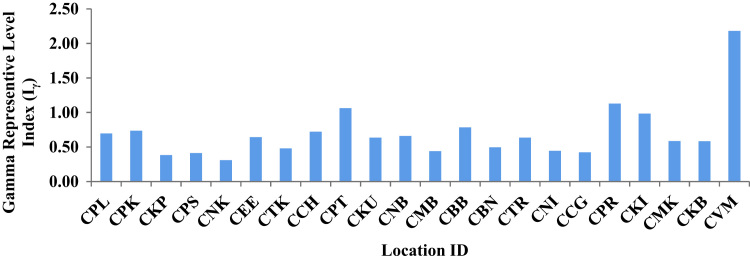
Location ID Vs Gamma Representative Level Index (*I*_γ_).

In order to examine whether the samples meet these limits of dose criteria, another radiation hazard index, the gamma representative level index (Iγ), is used to estimate the level of gamma radiation hazard associated with the natural radionuclides in specific investigated samples. It is used only as a screening tool for identifying materials that might become health concerns when used as construction materials [6]. The index was evaluated by the following equation [7]:

(2) $$I\gamma =\frac{A_{U}}{150}+\frac{A_{\mathrm{Th}}}{100}+\frac{A_{K}}{1500\;}$$

where *A*_U_, *A*_Th_ and *A*_K_ are the specific activities of ^238^U, ^232^Th and ^40^K (Bq kg^−1^) in respectively. Values of Iγ ≤ 1 correspond to an annual effective dose of less than or equal to 1 mSv, while Iγ ≤ 0.5 corresponds to an annual effective dose less than or equal to 0.3 mSv [8].The calculated values (Table 1) vary from 0.3106(Nettukuppam) – 2.1822(Vadanemmeli) with an average of 0.7016. The most of the studied locations did not exceed the recommended upper limit of unity indicating that the hazardous effects of these radiations are negligible. Fig. 3 shows the locations and gamma representative level index (Iγ).

## 2 Experimental design, materials and methods

### 2.1 Study area description

The study area, which spans from Pulicat Lake to Vadanemmeli of Chennai Coast, Tamil Nadu, India is located in one of the most populated regions of southeastern, India. The area is dominated by intensive industrial activities in which the discharge of their effluents into the river estuaries like Koratalliyar, Kosisthaliyar forms Ennore estuary surrounded by an major industrial corridor, Kuvam river loaded with Chennai city sewage, Adyar River forms Adyar estuary, Buckingham canal with untreated sewage traversing all these rivers has been going on for a long time. This coast is a very important environmental (Comprises biosphere reserve at Pulicat Lake, Bird sanctuary, Mangroves in Ennore, Adyar and Kuvam estuaries, Muttukkaadu backwater), economical, commercial, agricultural and recreational location in southeastern India. This study was performed to determine the impacts of radiation hazard associated with sediments collected in and around Chennai coast along the East Coast of Tamil Nadu and to assess the radiation hazards due to concentration of natural radionuclides.

### 2.2 Sample collection and preparation

**Fig. 4:**
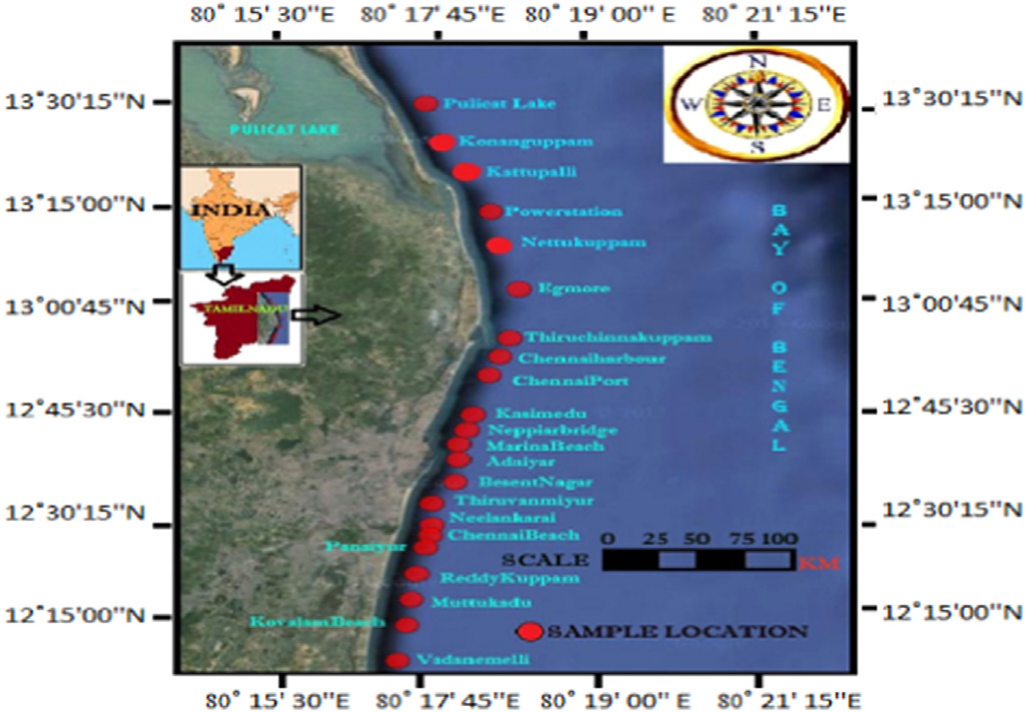
Location Map of the Study Area.

Totally 22 sediment samples were collected from Pulicat Lake to Vadanemmeli of Chennai coast along the Bay of Bengal Coastline in Southeastern Tamil Nadu, India using a Peterson grab sampler from 10 m water depths during the pre-monsoon season [9]. The sampling team initially approach to the beach by road and coastal craft hired from artisanal fishermen conveyed them to a sampling point after 45 min of sailing was utilized for sample collection. All sampling points were located parallel to the shoreline as shown in Fig. 4. The inter station spacing was maintained at 3 Knots for the study area.

The Peterson grab sampler is suitable for sampling nearshore seabed sediments particularly, in locations, where the sea bed is dominated by sandy, silt and/or gravelly sediments. This technique is the conventional method of sampling shallow sea bottom sediments [10], [11], [12]. The grab sampler collects the sediment samples at 25 cm below the seabed in all sampling points. From the grabbed samples, 10 cm thick sediment layer was scooped out from the middle of the grab to avoid metal contamination by the jaws of the grab.

Table 2 shows the geographic coordinates (latitudes and longitudes) of the various sampling locations. A handheld Global Positioning System (GPS) used for measuring the coordinates of the sampling points. Each sediment sample was carefully taken from the central portion of the craft and dredged with a plastic spatula previously washed with 2 M HCl and 2 M HNO_3_ to avoid any contamination [13]. The samples were stored in plastic bags at 4 °C. Generally sediments are refrigerated at < 6 °C to determine the concentration of bioavailable metals using cooling box with ice or portable refrigerator. Hence, in the present study samples were refrigerated at 4 °C using cooling box with ice and brought to laboratory. Then samples were air-dried and larger stone fragments or shells were removed by hand picking.

**Table 2:** Latitude and Longitude values of Study Area.

**S. No**	**Name of the Location**	**Sample ID**	**Latitude**	**Longitude**
1	Pulicat Lake	CPL	13°34'3.82"N	80°18'0.75"E
2	Pulicat (Koonangkuppam)	CPK	13°25'31.42"N	80°21'26.12"E
3	Kattupalli	CKP	13°19'27.33"N	80°22'51.77"E
4	Powerstation	CPS	13°15'35.37"N	80°22'21.94"E
5	Nettukuppam	CNK	13°14'10.50"N	80°21'53.23"E
6	Ennore	CEE	13°12'41.88"N	80°21'18.71"E
7	Tiruchinnakuppam	CTK	13° 9'36.02"N	80°20'32.34"E
8	Chennai Harbor (Nagooranthottam)	CCH	13° 8'20.61"N	80°20'8.02"E
9	Chennai Port (Kasimedu Fishing Harbour)	CPT	13° 6'5.45"N	80°19'44.78"E
10	Kasimedu-Tondiarpet	CKU	13° 7'14.61"N	80°19'44.04"E
11	Neppiarbridge	CNB	13° 4'17.77"N	80°19'34.47"E
12	Marina Beach	CMB	13° 2'34.23"N	80°18'20.02"E
13	Broken Beach(Adaiyaralamaram)	CBB	13° 0'54.40"N	80°18'21.48"E
14	Besentnagar	CBN	13° 0'8.21"N	80°18'17.37"E
15	Thiruvanmiyur	CTR	12°59'8.39"N	80°18'0.98"E
16	Neelankarai	CNI	12°57'2.18"N	80°17'29.61"E
17	Chennai Golden Beach	CCG	12°55'3.90"N	80°17'16.44"E
18	Panaiyur	CPR	12°53'2.32"N	80°17'4.18"E
19	Kanathursunami, (Reddykuppam)	CKI	12°50'12.66"N	80°16'34.01"E
20	Muttukaadu (Karikattukuppam)	CMK	12°48'36.74"N	80°16'40.72"E
21	Kovalam Beach	CKB	12°47'24.36"N	80°16'48.33"E
22	Vadanemmeli, (Puthiyakalpakkam)	CVM	12°44'59.05"N	80°16'39.20"E

Then the samples were air dried at 105 °C for 24 h to a constant weight and sieved through 250 μm mesh [9]. The homogenized sample was placed in a 250 g air tight PVC container to avoid radon or thorn escaping from the container. The inner lid was placed and closed tightly with outer cap. Each sediment sample container was left for at least 5 weeks to reach secular equilibrium between radium and thorium, and their progenies [14].

### 2.3 Gamma spectrometric analysis

Sediment samples were subjected to gamma spectral analysis with a counting time of 20,000 s. A 3 × 3 NaI(Tl) detector was employed with adequate lead shielding which reduced the background by a factor of about 95%. The concentrations of various radionuclides of interest were determined in Bq kg^−1^ using the count spectra. The energy calibration is required for various energies of radionuclides in the measurement of activity concentrations for the detector geometry size and selected samples. As the measurement is for the natural radioactive elements ^40^K, uranium and thorium, the gamma energies selected are 1460 keV for ^40^K, 1763 keV (from daughter product ^214^Bi) for uranium and 2614 keV (from daughter product ^208^Tl) for thorium [15]. The detection limit of NaI(Tl) detector system for ^40^K, ^238^U and ^232^Th are 8.50, 2.21 and 2.11 Bq kg^–1^ respectively for a counting time of 20, 000 seconds.
